# The significance of highlighting the oestrogen receptor low category in breast cancer

**DOI:** 10.1038/s41416-020-1009-1

**Published:** 2020-07-27

**Authors:** Ivan K. Poon, Julia Y. Tsang, Joshua Li, Siu-Ki Chan, Ka-Ho Shea, Gary M. Tse

**Affiliations:** 1 Department of Anatomical and Cellular Pathology, Prince of Wales Hospital, The Chinese University of Hong Kong, Ngan Shing Street, Shatin, NT Hong Kong; 2 Department of Pathology, Kwong Wah Hospital, Yau Ma Tei, Hong Kong; 3 Department of Pathology, Tuen Mun Hospital, Tuen Mun, Hong Kong

**Keywords:** Oncology, Breast cancer

## Abstract

The latest ASCO/CAP guideline has recommended to report oestrogen receptor (ER) low cases (ER^lo^; 1–10%) as “ER low positive category”, prompting us to compare the clinicopathologic features, biomarkers, survival and treatment of the ER^lo^ cases with other subgroups (ER negative (ER^neg^) and ER high (ER^hi^)). ER^lo^ cases revealed more similar clinicopathologic and biomarker profiles (including younger age, larger tumour, high proliferation, HER2 and basal markers expression) to ER^neg^ than ER^hi^ cancers. The ER^lo^ cases receiving hormonal therapy showed a similarly poor outcome as ER^neg^ cancers. However, majority of ER^lo^ cases were downstaged to stage I in the 8th AJCC pathological prognostic staging, highlighting a risk of potential under treatment. Overall, our data highlighted the differences of ER^lo^ from other ER^pos^ cases and their management should be considered separately.

## Background

Oestrogen receptor (ER) expression as assessed by immunohistochemistry is an important predictive marker for hormonal therapy (HT) in breast cancers. Most guidelines set a positive threshold at 1% or above of any staining intensity and such patients are eligible for HT. However, there is a concern that the cases with low ER expression (ER^lo^, i.e. 1–10%) may be biologically distinct from the high ER expressing cases (ER^hi^, i.e. >10%) and not benefit from HT.^1^ The latest ASCO/ CAP guideline has acknowledged this and recommended reporting ER^lo^ tumours as “ER Low Positive” category.^2^ A standardised comment is suggested to acknowledge the limited data on the overall benefit of HT for ER^lo^ patients, the heterogeneity in their behaviour and biology as well as their similarity to ER-negative (ER^neg^) cancers in gene profiling. Decision regarding appropriate treatment should be made after considering the totality of the information about the individual case. Here, we re-evaluated the characteristic of these ER^lo^ cases in comparison with ER^neg^ and ER^hi^ cases and the relevance of the new ER^lo^ category in breast cancer management.

## Methods

The study included 1824 consecutive cases of breast cancer diagnosed between 2002 and 2009 from three involved hospitals (Prince of Wales Hospital, Kwong Wah Hospital and Tuen Mun Hospital). Baseline demographic, clinical, treatment and outcome information were retrieved from the medical records. All patients were managed according to the NCCN guidelines. Histologic features including grade, necrosis and stromal tumour infiltrating lymphocytes (sTIL) were assessed on H&E slides as described previously.^3^ Immunohistochemical data on routine breast markers (including ER, progesterone receptor (PR), HER2 and Ki67), basal markers (CK5/6, CK14, EGFR, p63 and c-Kit) and androgen receptor (AR) were retrieved from our previous tissue microarray analysis.^3^ ER expression was also verified with medical records, and all cases were categorised into ER^neg^ (0%), ER^lo^ (1–10%) and ER^hi^ (>10%). Disease‐free survival (DFS) and breast-cancer-specific survival (BCSS) were analysed using the Kaplan–Meier method and compared between groups using the log‐rank test. Comparison of features between different ER groups was performed using Chi‐square or Fisher’s exact test. All statistical analyses were performed in SPSS version 23.

## Results

Table 1 summarised the clinical, histologic and biomarker features of the cohort. Fifty-four cases (3%) were ER^lo^, 503 (27.6%) cases were ER^neg^ and 1266 (69.4%) cases were ER^hi^. Compared to ER^hi^ cases, ER^lo^ cases were associated with larger tumour, higher grade, more necrosis, more sTIL, higher pN stage, high KI67, HER2, EGFR and CK5/6 positivity but PR and AR negativity (*p* ≤ 0.039). Among the ER^lo^ cases, only 24 cases (46.0%) had high expression of PR, much lower than the 73.7% (928/1259) in ER^hi^ cases. Compared to ER^neg^ cases, ER^lo^ cases were associated with higher PR but lower grade, lower expression of CK5/6 and CK14 (*p* ≤ 0.014). ER^lo^ cases were associated with more LVI (*p* = 0.024 and 0.011, respectively) and younger age (*p* = 0.005 and 0.004, respectively) than both ER^neg^ and ER^hi^ groups.

**Table 1:** Correlation of ER expression with clinicopathologic features and biomarkers.

	ER				*p*-value		
	Negative	Low	High	Total	All	Neg vs lo	Lo vs hi
*Clinicopathologic features*							
Age					0.014	0.005	0.004
Median	52	47	52	51			
IQR	45–61	44–54	45–62	45–61			
Range	23–101	22–82	27–97				
Tumour size					<0.001	0.782	0.003
Median	2.5	2.5	2.1	2.3			
IQR	2.0–3.5	2.1–6.7	1.5–3.0	1.6–3.3			
Range	0.1–13.0	1.1–8.0	0.1–10.2				
Grade					<0.001	0.014	<0.001
1	13 (2.6%)	3 (5.6%)	228 (18.0%)	244 (13.4%)			
2	99 (19.7%)	17 (31.5%)	641 (50.6%)	757 (41.5%)			
3	391 (77.7%)	34 (63.0%)	398 (31.4%)	823 (45.1%)			
Total	503	54	1267	1824			
Necrosis					<0.001	0.282	<0.001
Neg	270 (54.9%)	32 (62.7%)	1082 (87.8%)	1384 (77.9%)			
Pos	222 (45.1%)	19 (37.3%)	151 (12.2%)	392 (22.1%)			
Total	492	51	1233	1776			
LVI					0.040	0.024	0.011
Neg	355 (74.3%)	31 (59.6%)	913 (75.3%)	1299 (74.5%)			
Pos	123 (25.7%)	21 (40.4%)	300 (24.7%)	444 (25.5%)			
Total	478	52	1213	1743			
sTIL					<0.001	0.738	0.001
Low (>20%)	278 (68.0%)	32 (71.1%)	958 (89.3%)	1268 (83.0%)			
High (≤20%)	131 (32.0%)	13 (28.9%)	115 (10.7%)	259 (17.0%)			
Total	409	45	1073	1527			
Histotype					0.018	0.429	0.050
IDC-NOS	439 (87.3%)	44 (81.5%)	1148 (90.6%)	1631 (89.4%)			
Non-IDC-NOS^a^	64 (12.7%)	10 (18.5%)	119 (9.4%)	193 (10.6%)			
Total	503	54	1267	1824			
pN					0.009	0.064	0.015
0	255 (51.7%)	16 (30.8%)	618 (51.0%)	889 (50.6%)			
1	132 (26.8%)	21 (40.4%)	353 (29.1%)	506 (28.8%)			
2	53 (10.8%)	10 (19.2%)	156 (12.9%)	219 (12.5%)			
3	53 (10.8%)	5 (9.6%)	84 (6.9%)	142 (8.1%)			
Total	493	52	1211	1756			
pT					<0.001	0.841	0.012
1	167 (33.6%)	14 (25.9%)	591 (47.4%)	772 (42.9%)			
2	274 (55.1%)	35 (64.8%)	583 (46.7%)	892 (49.6%)			
3	38 (7.6%)	5 (9.3%)	50 (4.0%)	93 (5.2%)			
4	18 (3.6%)	0 (0%)	24 (1.9%)	42 (2.3%)			
Total	497	54	1248	1799			
AS					<0.001	0.258	0.007
IA	97 (20.5%)	8 (16.0%)	346 (29.3%)	451 (26.5%)			
IB	2 (0.4%)	1 (2.0%)	15 (1.3%)	18 (1.1%)			
IIA	172 (36.4%)	10 (20.0%)	385 (32.7%)	567 (33.3%)			
IIB	79 (16.7%)	15 (30.0%)	188 (15.9%)	282 (16.6%)			
IIIA	62 (13.1%)	11 (22.0%)	153 (13.0%)	226 (13.3%)			
IIIB	13 (2.7%)	0 (0%)	11 (0.9%)	24 (1.4%)			
IIIC	48 (10.1%)	5 (10.0%)	81 (6.9%	134 (7.9%)			
Total	473	50	1179	1702			
PPS					<0.001	<0.001	0.003
IA	51 (10.8%)	15 (30.6%)	635 (54.0%)	701 (41.3%)			
IB	54 (11.4%)	12 (24.5%)	241 (20.5%)	307 (18.1%)			
IIA	167 (35.4%)	7 (14.3%)	118 (10.0%)	292 (17.2%)			
IIB	40 (8.5%)	9 (18.4%)	69 (5.9%)	118 (7.0%)			
IIIA	74 (15.7%)	2 (4.1%)	57 (4.9%)	133 (7.8%)			
IIIB	31 (6.6%)	4 (8.2%)	44 (3.7%)	79 (4.7%)			
IIIC	55 (11.7%)	0 (0%)	11 (0.9%)	66 (3.9%)			
Total	472	49	1175	1696			
HT					<0.001	<0.001	<0.001
No	329 (85.5%)	17 (34.0%)	53 (5.2%)	399 (27.6%)			
Yes	56 (14.5%)	33 (66.0%)	957 (94.8%)	1046 (72.4%)			
Total	385	50	1010	1445			
CT					<0.001	0.135	0.002
No	91 (23.3%)	7 (14.0%)	346 (35.2%)	444 (31.2%)			
Yes	299 (76.7%)	43 (86.0%)	637 (64.8%)	979 (68.8%)			
Total	390	50	983	1423			
Relapse					<0.001	0.848	0.025
No	333 (76.2%)	39 (75.0%)	954 (86.2%)	1326 (83.1%)			
Yes	104 (23.8%)	13 (25.0%)	153 (13.8%)	270 (16.9%)			
Total	437	52	1107	1596			
BCSS					<0.001	0.530	0.082
No	310 (79.5%)	40 (83.3%)	876 (90.9%)	1226 (87.4%)			
Yes	80 (20.5%)	8 (16.7%)	88 (9.1%)	176 (12.6%)			
Total	390	48	964	1402			
*Biomarkers*							
Ki67					<0.001	0.329	0.004
Low (<20%)	224 (44.9%)	28 (51.9%)	875 (69.6%)	1127 (62.6%)			
High (≥20%)	275 (55.1%)	26 (48.1%)	383 (30.4%)	674 (37.4%)			
Total	499	54	1258	1801			
HER2^b^					<0.001	0.358	<0.001
Neg	321 (64.1%)	38 (70.4%)	1134 (90.1%)	1493 (82.3%)			
Pos	180 (35.9%)	16 (29.6%)	125 (9.9%)	321 (17.7%)			
Total	501	54	1259	1814			
EGFR^c^					<0.001	0.118	<0.001
Neg	442 (88.8%)	44 (81.5%)	1238 (98.8%)	1724 (95.5%)			
Pos	56 (11.2%)	10 (18.5%)	15 (1.2%)	81 (4.5%)			
Total	498	54	1253	1805			
PR					<0.001	<0.001	<0.001
Neg	409 (82.1%)	13 (25.0%)	156 (12.4%)	578 (32.0%)			
1–20%	54 (10.9%)	15 (28.8%)	175 (13.9%)	244 (13.5%)			
>20%	35 (7.0%)	24 (46.2%)	928 (73.7%)	987 (54.6%)			
Total	498	52	1259	1809			
C-kit^c^					<0.001	0.133	0.227
Neg	384 (77.1%)	44 (86.3%)	1141 (91.2%)	1569 (87.2%)			
Pos	114 (22.9%)	7 (13.7%)	110 (8.8%)	231 (12.8%)			
Total	498	51	1251	1800			
P63^c^					<0.001	0.787	0.229
Neg	459 (92.2%)	50 (94.3%)	1216 (97.0%)	1725 (95.6%)			
Pos	39 (7.8%)	3 (5.7%)	38 (3.0%)	80 (4.4%)			
Total	498	53	1254	1805			
CK5/6^c^					<0.001	0.005	0.039
Neg	343 (68.9%)	47 (87.0%)	1176 (94.0%)	1566 (86.9%)			
Pos	155 (31.1%)	7 (13.0%)	75 (6.0%)	237 (13.1%)			
Total	498	54	1251	1803			
CK14^c^					<0.001	0.005	1.00
Neg	424 (85.0%)	52 (98.1%)	1220 (97.4%)	1696 (94.0%)			
Pos	75 (15.0%)	1 (1.9%)	33 (2.6%)	109 (6.0%)			
Total	499	53	1253	1805			
AR^d^					<0.001	0.146	<0.001
Neg	240 (75.2%)	31 (86.1%)	323 (41.7%)	594 (52.6%)			
Pos	79 (24.8%)	5 (13.9%)	451 (58.3%)	535 (47.4%)			
Total	319	36	774	1129			

^a^Non-IDC-NOS included 50 IDC with medullary-like features, 43 invasive lobular carcinoma, 25 mucinous carcinomas, 16 metaplastic carcinoma, 16 micropapillary carcinoma and 43 other miscellaneous subtypes (including pleomorphic ILC, neuroendocrine tumour, signet carcinoma, tubular carcinoma, papillary carcinoma, etc).

^b^IHC 3+ according to ASCO guideline was considered HER2+.

^c^5% was used as cutoff.

^d^1% was used as cutoff.

Among the cases with treatment data, 14.5% (56/385), 66.0% (33/50) and 94.8% (957/1010), respectively, of the ER^neg^, ER^lo^ and ER^hi^ cases received HT while 96.8% (299/309), 86.0% (43/50) and 64.8% (637/983) received chemotherapy, respectively. Most ER^neg^ cases received HT were PR^pos^ and some others had coexisting ER^pos^ DCIS. Some ER^lo/hi^ cases did not receive HT because of the higher ER diagnostic threshold applied for the earlier cases, patients’ comorbidity and refusal of treatment. Compared to ER^hi^ cases, more ER^lo^ cases received chemotherapy (*p* = 0.002), but less received HT (*p* < 0.001). Follow-up information was available for 1597 patients (median follow-up of 73 months, range 1–210 months). Among them, 3.1% and 66.1% have follow-up of ≤1 year and >5 years, respectively. The rate of relapse/mortality of ER^lo^ cases (25.0%) was higher than ER^hi^ cases (13.8%), but similar to ER^neg^ cases (23.8%) (Table 1). Notably, high sTIL level showed a reduced mortality rate in both ER^lo^ (low Vs high sTIL: 9.1 Vs 24.1%) and ER^neg^ (14.4 Vs 24.0%) cancers. Additionally, the DFS of ER^lo^ cases was significantly worse than ER^hi^ cases (log-rank = 5.884, *p* = 0.015), but comparable to ER^neg^ cases (log-rank = 0.020, *p* = 0.887) (supplementary Figure). Among patients receiving HT, those with ER^lo^ cancers showed a significantly worse outcome than the ER^hi^ cancer patients, but no difference from those ER^neg^ cancer patients (without HT). If AJCC staging criteria were applied, 37 of the 49 (75.5%) ER^lo^ cases would be downstaged by at least one substage (17 cases with ≥2 substages), and more ER^lo^ cases were in pathological prognostic staging (PPS) stage I (55.1% (27/49)) compared to 18.0% stage I in anatomical staging (AS), and this may pose significant prognostic implication. Among all the PPS IA cases in the current cohort, cases of ER^lo^ showed a significantly poorer DFS than ER^hi^ cases (log-rank = 3.849, *p* = 0.050) (supplementary Figure).

## Discussion

Here, we observed a similar proportion of ER^lo^ cases as reported from a large series,^4^ but lower than some other smaller series.^5,6^ The ER^lo^ cases, as reported,^4,6^ showed distinct clinicopathologic and biomarker profiles from ER^hi^ cases. They were associated with expression of some basal markers, albeit at a lower rate than ER^neg^ cases, and this was concordant with previous report that most ER^lo^ cases were of basal molecular subtype.^6^ Thus, ER^lo^ cases were heterogeneous, with some cases similar to ER^neg^ cancers. Little apparent benefit from adjuvant HT for patients with low ER has been suggested.^7^ For treatment, it has been speculated that interventions for ER^neg^ tumours may be appropriate for some ER^lo^ cases. In fact, ER^lo^ cases could behave clinically like triple negative breast cancer (TNBC) in terms of pathological complete response to neoadjuvant chemotherapy.^8^ Recently, immune blockade has been approved for treating TNBC.^9^ Despite the traditional view of ER^pos^ cancers as non-immunogenic, the enrichment of sTIL and the associated reduced mortality within ER^lo^ cases may suggest an active role of antitumour immunity and the potential of immunotherapy.

Recently, ER has been incorporated into the AJCC PPS together with PR, HER2 and grade. PPS shows a superior prognostication power than the traditional TNM AS.^10^ In the PPS, compared to the corresponding AS, if a breast cancer expresses ER and/or HER2, it will be downstaged. There is, however, a caveat as in the AJCC guideline, ER positivity was defined as expression in 1% or more of the tumour cells, without segregation into ER^lo^ and ER^hi^. As we demonstrated, ER^lo^ cases were biologically more similar to ER^neg^ and showed worse survival in the downstaged cases. Thus, using the approach in AJCC staging, there is a real risk of downstaging ER^lo^ cases that behave more like ER^neg^ cases biologically, resulting in potentially under treatment.

This analysis confirmed that distinct behaviour of ER^lo^ tumours, which are heterogeneous, with some resembling ER^neg^ tumours biologically (higher likelihood of being basal-like, and worse prognosis), thus lending support to the new ASCO guideline. Furthermore, downstaging as per AJCC guideline for ER^lo^ cases may incur a real possibility of risk underestimation and under treatment.

Limitations of the analysis included retrospective nature of the study and the small number of ER^lo^ cases, which limited the power of statistical analysis. Moreover, some IHC data were obtained from TMA analysis. For PPS staging, no results from OncotypeDX were available.

## Supplementary information

supplementary figure

## Data Availability

The dataset used and analysed in the current study is available from the corresponding author on reasonable request.
