# Supplementary material for: The significance of highlighting the oestrogen receptor low category in breast cancer
Source: Br J Cancer. 2020 Jul 27;123(8):1223–7. doi: 10.1038/s41416-020-1009-1 (PMC7555863; doi:10.1038/s41416-020-1009-1)

Supplementary figure Kaplan Meier analysis of survival of different ER expressing subgroups in the overall cohort (upper); patients with HT (middle) and pathological prognostic stage IA cases (lower).


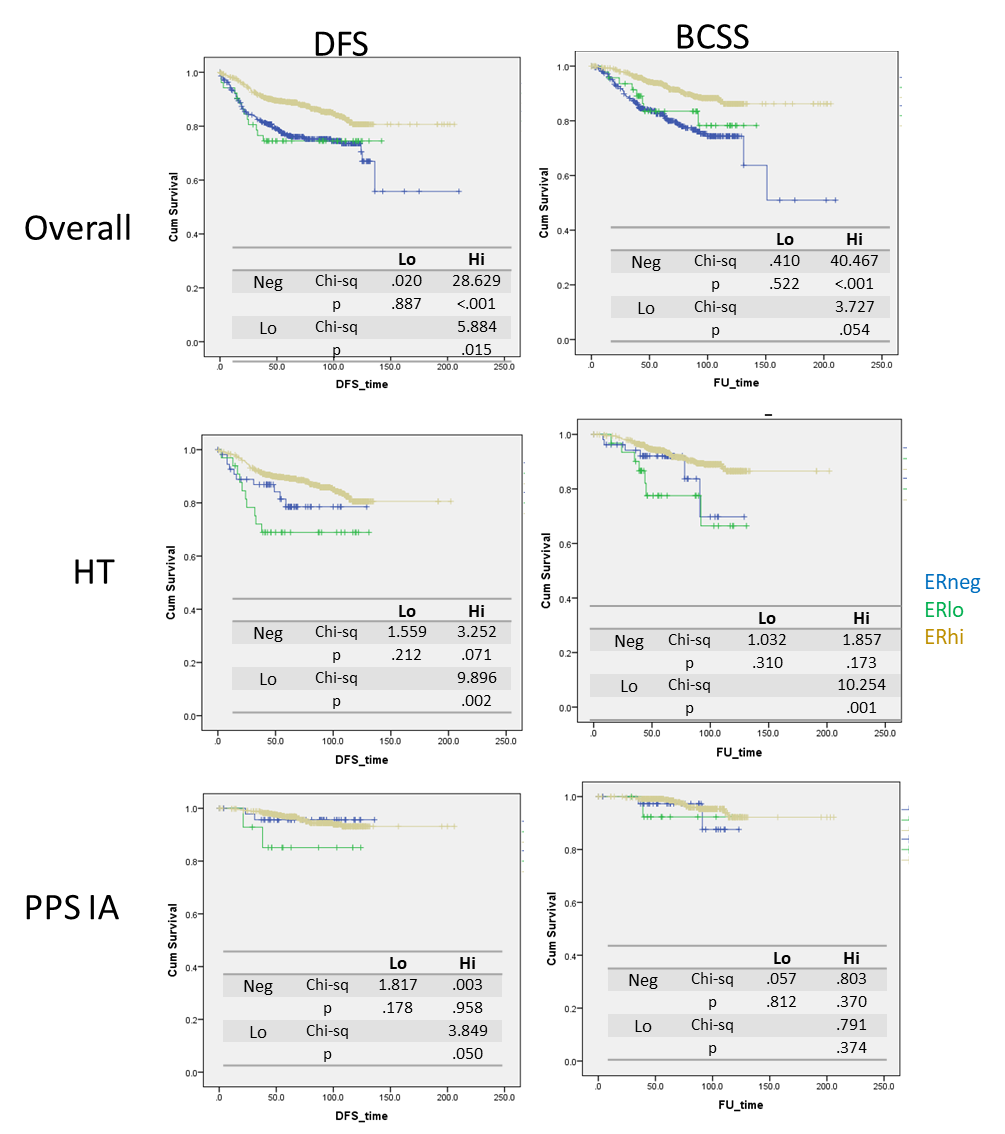

Supplement: Supplementary file 1 — supplementary figure [file 41416_2020_1009_MOESM1_ESM.docx]
